# Investigation of the Relationship between Apolipoprotein E Alleles and Serum Lipids in Alzheimer’s Disease: A Meta-Analysis

**DOI:** 10.3390/brainsci13111554

**Published:** 2023-11-06

**Authors:** Huaxue Xu, Jiajia Fu, Risna Begam Mohammed Nazar, Jing Yang, Sihui Chen, Yan Huang, Ting Bao, Xueping Chen

**Affiliations:** 1 Department of Neurology, West China Hospital, Sichuan University, Chengdu 610041, China; xuxuhuaxue@sina.com (H.X.); fujiajia0421@foxmail.com (J.F.); begamrisna@gmail.com (R.B.M.N.); yo_screw1900@163.com (J.Y.); chensihuiyyds@163.com (S.C.); 2 Management Center, West China Hospital, Sichuan University, Chengdu 610041, China; huangyanhy513@163.com (Y.H.); baoting199@163.com (T.B.)

**Keywords:** apolipoprotein E, serum lipids, Alzheimer’s disease, meta-analysis

## Abstract

Prior studies have yielded mixed findings concerning the association between apolipoprotein E(*APOE*)-*ε4* and serum lipids in patients with Alzheimer’s disease (AD) and healthy individuals. Some studies suggested a relationship between *APOEε4* and serum lipids in patients with AD and healthy individuals, whereas others proposed that the *APOEε4* allele affects lipids only in patients with AD. Our study aimed to investigate whether *APOE* alleles have a distinct impact on lipids in AD. We conducted a comprehensive search of the PubMed and Embase databases for all related studies that investigate *APOE* and serum lipids of AD from the inception to 30 May 2022. Elevated total cholesterol (TC) and low-density lipoprotein (LDL) levels were found in *APOEε4* allele carriers compared with non-carriers. No significant differences were found for high-density lipoprotein (HDL) and triglyceride (TG) levels in *APOEε4* allele carriers compared to non-carriers. Notably, elevated TC and LDL levels showed considerable heterogeneity between patients with AD and healthy controls. A network meta-analysis did not find a distinct effect of carrying one or two *APOEε4* alleles on lipid profiles. Higher TC and LDL levels were found in *APOEε4* allele carriers compared with non-carriers, and the difference was more significant in patients with AD than in healthy controls.

## 1. Introduction

Alzheimer’s disease (AD) presents as a prevalent progressive neurodegenerative disease characterized by an insidious onset, progressive memory decline, cognitive impairment, and a spectrum of behavioral and psychological symptoms [1]. The development of AD appears to be a result of the complex interplay of genetic and environmental factors [2], hence rendering effective treatment of AD a formidable challenge [3]. The multifactorial etiology of this global health challenge has driven many research endeavors to unravel the complex web of causative elements of AD. Among these factors are apolipoprotein E (*APOE*) and its allelic variants, specifically the *APOE ε4* allele, which have emerged as being noteworthy.

The human APOE gene is encoded on chromosome 19, and it has three allelic variants: *ε2*, *ε3*, and *ε4* [4]. Notably, the individuals carrying the *APOEε4* allele exhibit a high risk of sporadic AD [5]. Individuals with a single *APOEε4* allele have a 3.2 times higher risk of developing AD, whereas, in those with two *APOEε4* alleles, the risk of developing AD is increased by 8 to 10 folds [6]. This can be attributed to the influence of the *APOEε4* allele on amyloid-β (Aβ), either by reducing its clearance or by increasing its production in the brain [7]. 

In neuroimaging investigations of APOE polymorphism in healthy individuals, there has been a predominant focus on examining gray matter alterations in middle or late life, particularly in brain regions associated with significant AD pathological findings. Even in individuals showing no clinical symptoms, documentation has shown a reduction in the gray matter within the hippocampal and frontotemporal regions in *APOEε4* allele carriers compared with non-carriers [8].

Moreover, the human *APOE* allele encodes a polyclonal lipoprotein integral to metabolic processes, including cholesterol transport [9]. Although *APOE* alleles have a certain impact on lipid profiles [10,11,12,13,14], current research results are inconsistent [11,14]. Some studies have identified elevated levels of low-density lipoprotein (LDL) and total cholesterol (TC) in *APOEε4* allele carriers (*APOEε4* allele-C) compared with non-carriers (*APOEε4* allele-N), whereas others [10,12] have reported the opposite. Furthermore, some studies [13,15] have reported that significant differences exist in high-density lipoprotein (HDL) levels between carriers and non-carriers of the *APOEε4* allele. However, such distinctions were not observed in other studies [12,13]. Intriguingly, no systematic analyses have focused on the differences in lipid profiles between single *APOEε4* allele carriers and *APOEε4* homozygous individuals concerning lipid profiles.

Most researchers believe that lipid metabolism is very important in the pathophysiological mechanism of AD [16]. Notably, the latest meta-analysis summarized the disparities in lipid profiles between individuals with AD and healthy controls [17]. Since the *APOEε4* allele affects both lipid metabolism and the pathophysiology of AD, it has been hypothesized that the special relationship between the *APOEε4* allele and lipid metabolism is unique in AD. Some studies have found a relationship between the *APOEε4* allele and lipid profiles in patients with AD and healthy control populations [18], whereas others have discerned this association exclusively within the AD population [13]. 

Additionally, most meta-analyses summarized the differences in lipids between patients with AD and healthy controls, but there has been no relevant summary analysis that has explored whether the unique relationship between the *APOEε4* allele and lipids differs between patients with AD and healthy controls. Therefore, we systematically compared the lipid profiles between carriers and non-carriers of the *APOE4* allele among patients with AD and healthy controls and investigated whether the effect of *APOE* on lipids is unique in AD. We hypothesized that the *APOEε4* allele might cause the development of AD by influencing lipid metabolism.

## 2. Materials and Methods

### 2.1. Search Strategy

Two independent investigators searched the PubMed, Embase, Web of Science, and Chinese databases on 30 May 2022. The following medical subject heading (MeSH) terms and topic terms were used as the search terms: “Lipid”, “Cholesterol”, “Triglycerides”, “Alzheimer’s disease”, “Alzheimer Dementia”, “Apoprotein E”, and “*APOE*”. 

### 2.2. Inclusion and Exclusion Criteria

The inclusion criteria were as follows: (1) all articles that reported the results of *APOE* alleles and were grouped participants according to whether they carried the *APOEε4* allele and/or different *APOE* alleles; (2) articles reporting data as mean ± standard derivation (SD); (3) studies that analyzed patients with AD patients or healthy controls as the study populations; (4) studies that included patients diagnosed with AD; and (5) studies that included healthy controls with normal cognitive function and no neurological disease. 

The exclusion criteria were as follows: reviews, conference papers, letters, comments, editorials, case reports, and abstracts without an available full text. 

### 2.3. Data Extraction and Quality Evaluation

FJJ and YJ conducted the preliminary screening of titles and abstracts and then screened potentially relevant full texts according to the inclusion criteria. A third investigator verified all the data. From each study, we collected the following data: the sample size, publication year, and participant characteristics (age, number of participants, sex ratio, country, and Mini-Mental State Examination scores). Relevant information was extracted independently by two investigators and verified by a third investigator. The Newcastle–Ottawa Quality Assessment Scale (NOS Scale) was used to assess the quality of the included studies [19]. The total score on this scale is 9, and a score of ≥6 is acceptable.

### 2.4. Statistical Analysis

We performed a meta-analysis using Stata, version 15.0 software (StataCorp LLC., College Station, TX, USA) and used the standardized mean difference (SMD) to obtain aggregate effects. The random-effects model was used if there was significant heterogeneity between the included studies (the Cochrane *Q* test result and I^2^ statistic: I^2^ > 50% or *p* < 0.1). The *z*-test was used to determine the overall effect. We assessed heterogeneity using sensitivity, meta-regression, and subgroup analyses and evaluated the publication bias using Begg’s and Egger’s tests. The standardized effect size was compared between multiple groups using network meta-analysis, and related indicators of each group were compared using the cumulative ranking curve (SUCRA).

## 3. Results

### 3.1. Study Selection and Characteristics

**Figure 1:**
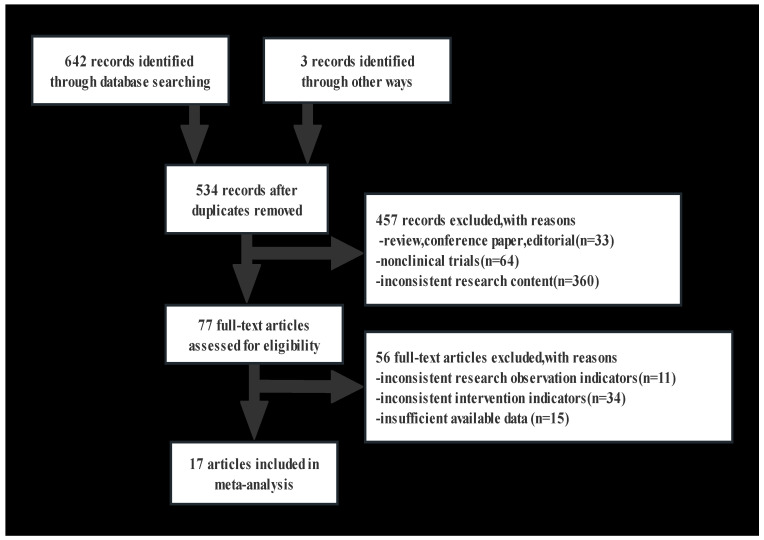
The literature screening flow chart.

The flow chart illustrates the systematic search and selection process (Figure 1); 17 studies were included in the final analysis [10,12,13,14,15,18,20,21,22,23,24,25,26,27,28,29,30] (Table 1). These selected studies, which were carefully evaluated for their relevance and contribution to our research objectives, are shown in Table 1. Eight of these studies grouped the participants on the basis of their APOEε4 allele status. Among them, four studies exclusively focused on individuals with AD, whereas the other four studies examined both patients with AD and healthy controls. APOE allele classification was further extended in six studies, which divided participants into three specific groups: *APOEε2* allele carriers, *APOEε3/3* carriers, and *APOEε4* allele carriers. Of these, two studies exclusively focused on the AD population, and the remaining four encompassed both AD and healthy control populations. The participants were divided into six subgroups based on their *APOE* alleles status across a total of five studies. Among them, one study was exclusively dedicated to the AD population, one study was exclusively dedicated to the control population, and three studies grouped both the AD and control populations. It is important to note that some studies did not analyze all pertinent variables, including but not limited to TC, triglycerides (TG), HDL, and LDL levels. These variances are essential to consider when interpreting the collective findings. The NOS Scale scores are shown in Table S1.

### 3.2. Data Extraction and Study Population 

We extracted data from eight articles focusing on TC and TG levels that included 652 individuals carrying *APOEε4* allele-C and 1038 individuals with *APOEε4* allele-N. Additionally, we collected information from seven articles regarding HDL and LDL levels that included 630 individuals carrying *APOEε4* allele-C and 987 individuals with *APOEε4* allele-N. 

### 3.3. Overall Effect, Heterogeneity, Publication Bias, and Subgroup Analysis

**Figure 2:**
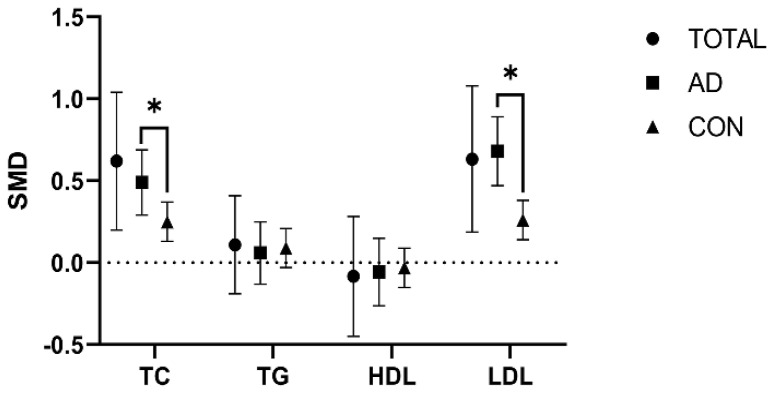
Comparison of the effect of the *APOEε4* allele on lipids in the AD and healthy control populations. The data are presented as the standardized mean difference (SMD) and 95% confidence interval. TOTAL: both the Alzheimer’s disease and healthy control populations; AD: Alzheimer’s disease population; CON: healthy control population; TC: total cholesterol; TG: triglycerides; HDL: high-density lipoprotein; LDL: low-density lipoprotein. *: *p* < 0.05.

To elucidate the overall effect across the studies, we used a random effect model to address the variances arising from differences present in the included studies. Noteworthy differences in TC and LDL levels were observed when comparing the APOE*ε4* allele-C and APOE*ε4* allele-N groups. Specifically, individuals in the *APOEε4* allele-C group showed higher TC and LDL levels than those in the *APOEε4* allele-N group (TC: SMD = 0.62 [0.2, 1.04], *p* = 0.004; LDL: SMD = 0.63 [0.9, 1.08], *p* = 0.005). However, studies indicated no difference in TG and HDL levels between those groups (TG: SMD = 0.08 [−0.19, 0.41], *p* = 0.108; HDL: SMD = −0.08 [−0.45, 0.28], *p* = 0.655) (Figure 2 and Figures S1–S4). 

Sensitivity analysis was conducted to identify the causes of heterogeneity and we were able to identify a clear cause of heterogeneity (Figures S5–S8). The funnel plot and bias test showed no significant publication bias (Figures S9–12, Tables S2–S5). 

However, subgroup analysis showed great heterogeneity in TC and LDL levels between the *APOEε4* allele-C and *APOEε4* allele-N groups among the AD and healthy control populations (TC: *p* = 0.042; LDL: *p* = 0.001). However, no heterogeneity was shown in HDL and TG levels (TG: *p* = 0.794; HDL: *p* = 0.823) (Figure 2 and Figures S13–S16). 

Notably, the AD and healthy control populations had elevated TC and LDL levels in the APOE*ε4* allele-C group compared with the APOE*ε4* allele-N group, but the degree of elevation was lower in the AD population than in the healthy control population (AD population: TC: SMD = 0.49 [0.29, 0.69], *p* = 0.000; LDL: SMD = 0.68 [0.47, 0.89], *p* = 0.000) (healthy control population: TC: SMD = 0.25 [0.13, 0.37], *p* = 0.000; LDL: SMD = 0.26 [0.14,0.38], *p* = 0.000) (Figure 2 and Figures S13–S16).

### 3.4. Comparison of the Lipids in APOEε3/ε3, APOEε2 Allele, and APOEε4 Allele Carriers

The network meta-analysis was performed to compare TC, TG, and LDL levels in individuals carrying different *APOE* alleles; *APOEε4* allele carriers had the highest SUCRA value, followed by *APOEε3/3* and *APOEε2* allele carriers, respectively (Tables S6–S8). However, regarding HDL levels, *APOEε4* allele carriers had the lowest SUCRA value, followed by *APOEε2* allele carriers, whereas *APOEε3/3* allele carriers had the highest SUCRA value (Table S9). 

### 3.5. Comparison of the Lipids between Six Groups of APOE Alleles

The network meta-analysis of six distinct groups formed by *APOE* alleles showed variations in the SUCRA values of TC levels as follows: *APOEε*3/*ε*4 > *APOEε4*/*ε*4 > *APOEε*3/*ε*3 > *APOEε*2/*ε*2 > *APOEε*2/*ε*4 > *APOEε*2/*ε*3 (Table S10). 

Similarly, SUCRA values of TG levels were as follows: *APOEε*2/*ε*2 > *APOEε*3/*ε*4 > *APOEε*3/*ε*3 > *APOEε4*/*ε*4 > *APOEε*2/*ε*3 > *APOEε*2/*ε*4 (Table S11). 

Owing to the limited availability of multiple data sets, sequencing comparisons of HDL and LD levels between these six groups could not be performed.

## 4. Discussion

### 4.1. Main Findings

We hypothesized that the *APOEε4* allele might cause the development of AD by influencing lipid metabolism. Studies have found that high levels of serum cholesterol are positively associated with an increased risk of dementia, and the prevalence of AD is reduced in patients taking cholesterol-lowering drugs [32]. A Mendelian randomization study of AD metabolism and risk confirmed the causal role of LDL, cholesterol, and serum total cholesterol in the high-risk of AD [33]. Some studies have found an association between blood lipids and Alzheimer’s disease, proving that blood lipids can be used as biomarkers for the early diagnosis of Alzheimer’s disease. It can also help predict the stage of prognosis and disease severity, and further studies are needed to find out the exact mechanisms behind these changes [34]. This study focused on the relationship between *APOE* alleles and serum lipid profiles, specifically TC, LDL, TG, and HDL levels in individuals with AD compared to healthy controls. Through our meta-analysis, we found that individuals carrying the *APOEε4* allele showed increased TC and LDL levels compared with those without the *APOEε4* allele. There was a statistically significant difference in TC levels between the *APOEε4* allele-C and *APOEε4* allele-N groups. The *p*-value indicated that the difference did not occur by chance and is, therefore, statistically significant. *APOEε4* allele-C carriers had higher LDL levels than non-*APOEε4* allele-C carriers.

Notably, no significant statistical differences were found in TG and HDL levels between these groups. These data reinforce the absence of statistically significant differences in TG and HDL levels between individuals with *APOEε4* allele-C and those without. Further analysis showed differences in TC and LDL levels between *APOEε4* allele-C and *APOEε4* allele-N groups with significant heterogeneity when considering AD and healthy controlled populations separately. These data suggest that in AD populations, TC and LDL levels are higher in *APOEε4* allele-C carriers than in *APOEε4* allele-N carriers, but the degree of elevation is lower than that seen in the healthy control populations. 

It is crucial to note that *APOEε4* acts as a main genetic risk factor for AD. Genome-wide association studies have shown that *APOEε4* is the strongest genetic risk factor for AD, irrespective of the age of onset [31].

### 4.2. APOE Functions in the Brain

The APOE gene encodes the APOE protein, which plays an important role in the transportation and metabolism of lipids [35]. APOE is responsible for the transportation of lipids and the maintenance of cholesterol homeostasis in the brain. It plays a crucial role in supplying neurons with cholesterol and facilitating the removal of excess cholesterol. It is also involved in other brain functions, such as promoting synaptic plasticity, transmitting signals, maintaining protein balance, modulating the immune system, and repairing after an injury [36].

### 4.3. APOE Isomers and Their Binding Specificity

Research has shown that the C-terminal domain of APOE is the key to lipoprotein binding and determines the specificity of APOE subtype lipidosis [37]. Specifically, *APOEε4* shows distinct characteristics, including poor lipidation compared with *APOEε2* and *APOEε3* alleles [38]. The *APOEε3* and *APOEε2* alleles prefer to bind to HDL, whereas the *APOEε4* allele prefers to bind to very low-density lipoprotein (VLDL) [39]. This variation in lipoprotein association is determined by differences in the interactions of the carboxyl-terminal domains among the isoforms, leading to *APOEε2* and *APOEε3* binding to smaller more phospholipid-enriched HDL, and *APOEε4* binding to larger triglyceride-rich VLDL [40].

### 4.4. Lipid-Binding Effects of APOEε 4 and Cholesterol Efflux

The lipid-binding features of *APOEε4* have substantial effects on the efflux of cholesterol and the metabolism of amyloid-beta (Aβ). The functional attributes of *APOE*, including receptor binding capabilities, molecular stability, and overall functionality, are conditional based on its lipidation status [41]. In vitro model studies have shown a pivotal role of lipidation in preventing self-aggregation of *APOE* [42]. Given the considerable influence of lipidation on many roles of *APOE*, it has been proposed as a potential therapeutic treatment for AD. Hence, there is the possibility to correct, as well as prevent, certain outcomes associated with neurodegeneration. The benefit of increasing lipidation and reducing lipid-free availability may offer greater advantages to the individuals who carry the *APOEε4* allele, which accounts for a larger percentage of both AD populations and healthy control populations [43]. 

Another complementary study observed that pharmacologically promoting cholesterol efflux can increase myelination in vitro and in vivo and improve cognition in APOE4/e-TR mice. This finding indicates a link between cholesterol dysregulation and myelination in *APOEε4* carriers, which may impact the onset and severity of cognitive decline in AD. Interventions such as pharmacological treatments, lifestyle, and dietary modifications aiming at restoring cholesterol equilibrium and myeline volume might help to increase the cognitive reserves in *APOEε4* carriers [44].

This proposal to augment APOE lipidation as a therapeutic approach shows the increasing understanding of the complex connection between lipid metabolism, APOE genetics, and AD pathogenesis. Further investigations are required to determine the practicality and effectiveness of using this approach in the clinical setting as a means to develop successful therapeutic interventions for AD.

Furthermore, the *APOEε4* allele has a strong lipid-binding affinity and a low recovery capacity, leading to impaired cholesterol efflux, culminating in an increased accumulation of cholesterol in cell membranes [45]. The distribution of elevated cholesterol levels on the plasma membrane of neurons correlated with increases in the metabolism of Aβ precursor protein (APP), which results in increased Aβ production [46]. In addition to neurons, astrocytes and microglia are also affected by impaired cholesterol efflux. In these cells, less cholesterol efflux reduces Aβ degradation, which may increase aggregation of Aβ into plaques [47]. 

### 4.5. HDL and Cholesterol Metabolism in APOE Non-Carriers

Longitudinal studies have shown that individuals with AD who are non-carriers of the *APOEε4* allele have elevated HDL levels. This elevation is associated with impaired cholesterol metabolism and impaired function, possibly resulting from reduced lipid availability in neuronal membranes [48]. Furthermore, in *APOEε4* allele non-carriers of AD-stratified populations, the enzyme 3-hydroxy-3-methylglutaryl-CoA synthetase was significantly associated with sporadic AD. This suggests potential cholesterol metabolic dysfunction in patients with AD who do not carry the *APOEε4* allele [49]. 

### 4.6. Heterogeneity and Implications in Clinical Practice

Subgroup analysis based on different populations yielded findings showing significant inter-group heterogeneity in patients with AD and the healthy controls, especially since the influence of the *APOEε4* allele on TC and LDL levels appears to be more pronounced in patients with AD than in the healthy control population. One important consideration is that TC and LDL in peripheral blood rarely enter the central nervous system (CNS). These lipids typically do not cross the blood–brain barrier in substantial amounts to cause harm to CNS function. Therefore, any effect of *APOE* alleles on peripheral TC and LDL levels may differ from their potential roles in the CNS. This raises an important question as to whether the influence of *APOE* alleles on peripheral lipid levels is related to the central pathological mechanism of AD. The exact nature of this relationship remains unclear, so it is an important area that warrants more comprehensive investigations. Therefore, more attention should be paid to AD in clinical practice and future studies, especially the lipid levels of patients with AD carrying the *APOEε4* allele.

Given the high degree of heterogeneity in this meta-analysis, we acknowledge that the exact cause of this variability has not been definitively identified despite performing sensitivity meta-regression and other analyses. We tried to exclude influential studies and found that heterogeneity could not be significantly reduced after re-analysis. This heterogeneity could be due to a combination of various factors, including different study populations, methodologies, and patient characteristics, such as age, sex, genetic background, medication use, ethnicity, and race. 

### 4.7. Sex-Based Analysis

Sex-based analysis can provide more insight into how sex-specific hormonal factors interact with *APOE* alleles to modulate lipid profiles and AD risk differently in men and women. There are significant differences between males and females in the regulation of fatty acid metabolism. Premenopausal women tend to have higher levels of polyunsaturated fatty acids than men [31], which may be due to higher estrogen levels affecting lipid metabolism in premenopausal women [50]. Additionally, women in premenopausal, menopausal transition states have alterations in various body fats, which are also related to changes in their estrogen concentrations [51]. Decreased estrogen levels in postmenopausal women can affect lipid metabolism, which increases the risk of cognitive decline [52].

Females with one copy of the *APOEε4* allele had about four times the risk of AD, whereas males with one copy of the *APOEε4* allele had only twice the risk [53]. It is unknown whether there are differences in lipid metabolism between different *APOEε4* allele groups with different sexes. A study conducted in 2022 showed that within the AD population, both sexes showed high levels of TC and LDL compared with the control group. Notably, among female patients with AD, TC and LDL levels were significantly higher in *APOEε4* allele carriers than in non-carriers. In contrast, the presence of the *APOEε2* allele was linked to reduced TC levels in male patients with AD compared with non-carriers. This particular influence was not evident among male controls, female controls, or female AD populations. However, further prospective studies are required to confirm these findings [54].

In our study, owing to insufficient data, it was not possible to conduct subgroup analysis based on sex, age, and medication use to explore various causes of heterogeneity. It is worth further exploring the sex-based differences in lipid metabolism between different *APOEε4* allele groups and how these differences can influence AD risk.

### 4.8. Dual APOE4 and Lipid Profiles

In addition, this study used network meta-analyses to explore the effect of both single and dual *APOEε4* alleles on lipid profiles. Interestingly, the presence of dual *APOEε4* alleles did not increase the degree of influence on lipid profiles compared with a single APOEε4 allele. This finding negates the notion that having a higher genetic predisposition (possessing two *APOEε4* alleles) leads to more lipid-related impacts in AD.

### 4.9. Comparisons with Other Studies and What This Study Added to the Existing Knowledge

In contrast to previous meta-analyses that primarily examined the differences in lipids between AD and healthy controls, this study took a more focused approach. We investigated the difference in lipid levels between those carrying *APOEε4* allele-C and *APOEε4* allele-N within the context of AD. Thus, we were able to evaluate the specific influence of the *APOEε4* allele on lipids in AD, which adds novel knowledge to improve understanding of the complex interplay between genetics and lipid metabolism in AD pathogenesis.

### 4.10. Study Strengths and Limitations

This study is the first comprehensive analysis of the distinctive relationship between the *APOEε4* allele and lipids in patients with AD and healthy controls. The influence of the presence of the *APOEε4* allele on blood lipids, and the differences between single and dual *APOEε4* allele lipids, were analyzed using MeSH terms in meta-analysis, which is the strength of this study. However, this study has some limitations. First, since the data on age and the sex ratio of the *APOEε4* allele carriers and the non-carriers were insufficient, we could not conduct a deeper subgroup analysis stratified by age and sex. Second, despite our best efforts to contact the respective authors, some articles had incomplete data.

## 5. Conclusions

This meta-analysis showed that *APOEε4* allele-C carriers had higher TC and LDL levels than *APOEε4* allele-N carriers, and the difference was significant between patients with AD and healthy participants. The dual *APOEε4* allele may not have an increased effect on the lipid profiles. The effect of dyslipidemia and interventions on lipids levels in AD, especially in *APOEε4* allele carriers, should be extensively studied in the future. Currently, there are no therapies targeting APOE for AD treatment. These studies offer new insights for potential future AD treatments and provide a basis for precision medicine. 

## Figures and Tables

**Table 1:** Details of the original studies included in the meta-analysis for *APOE* and AD.

Author-Year	Country	*n* (AD)	*n* (CON)	Sex (Male%) (AD)	Sex (Male%) (CON)	Age (AD)	Age (CON)	*APOE* (*n*)	Lipid Profiles (mmol/L)
Fernandes, 1999 [20]	Portugal	74	35	43.2	48.6	68.24 ± 9.02	64.97 ± 10.42	AD: *APOEε4*+(18), *APOEε4*−(27) CON: *APOEε4*+(4), *APOEε4*−(24) AD: *ε*2/*ε*2(0), *ε*2/*ε*3(3), *ε*2/*ε*4(0), *ε*3/*ε*3(24), *ε*3/*ε*4(13), *ε*4/*ε*4(5) CON: *ε*2/*ε*2(0), *ε*2/*ε*3(3), *ε*2/*ε*4(0), *ε*3/*ε*3(21), *ε*3/*ε*4(4), *ε*4/*ε*4(0)	TC, TG
Wehra, 2000 [21]	Poland	26	39	30.8	38.5	70.6 ± 7.3	70.0 ± 8.3	AD: *APOEε4*+(16), *APOEε4*−(10)	TC, TG, HDL, LDL
Sheng, 2000 [22]	China	39	40	54.8				AD: *ε*2+(2), *ε*3/*ε*3(20), *ε*4+(17)	TC, TG, HDL, LDL
Isbir, 2001 [12]	Turkey	35	29	25.7	70	73.91 ± 7.35	73.62 ± 13.63	AD: *APOEε4*+(7), *APOEε4*−(28)	TC, TG, HDL, LDL
Jingbin, 2002 [23]	China	109	98	41.3	54.1	3.7 ± 7.1	9.2 ± 6.5	AD: *ε*2/*ε*2(0), *ε*2/*ε*3(8), *ε*2/*ε*4(0), *ε*3/*ε*3(46), *ε*3/*ε*4(37), *ε*4/*ε*4(18) CON: *ε*2/*ε*2(1), *ε*2/*ε*3(14), *ε*2/*ε*4(1), *ε*3/*ε*3(73), *ε*3/*ε*4(8), *ε*4/*ε*4(1)	TC, TG
Xiangyu, 2002 [24]	China	48	84	64.6	73.8	73 ± 8	61 ± 1	AD: *ε*2+(10), *ε*3/*ε*3(23), *ε*4+(15) CON: *ε*2+(10), *ε*3/*ε*3(67), *ε*4+(5)	TC, TG
Al-Shammari, 2004 [25]	Kuwait		106		94.3		40.5 ± 4.7	CON: *ε*2/*ε*2(2), *ε*2/*ε*3(7), *ε*2/*ε*4(1), *ε*3/*ε*3(78), *ε*3/*ε*4(18), *ε*4/*ε*4(0)	TC, TG, HDL, LDL
Raygani, 2006 [13]	Iran	94	111	43.6	36.9	74.2 ± 10	72 ± 11.4	AD: *APOEε4*+(34), *APOEε4*−(60) CON: *APOEε4*+(14), *APOEε4*−(97)	TC, TG, HDL, LDL
Hall, 2006 [10]	India	29	1046					AD: *APOEε4*+(14), *APOEε4*−(15) CON: *APOEε4*+(416), *APOEε4*−(630)	TC, TG, HDL, LDL
Sabbagh, 2006 [15]	America	142				52–96		AD: *APOEε4*+(86), *APOEε4*−(60) AD: *ε*2/*ε*2(0), *ε*2/*ε*3(10), *ε*2/*ε*4(0), *ε*3/*ε*3(50), *ε*3/*ε*4(65), *ε*4/*ε*4(17)	TC, TG, HDL, LDL
Dongmei, 2008 [26]	China	77	158	59.7	55.7	3.3 ± 4.6	3.8 ± 5.0	AD: *ε*2+(4), *ε*3/*ε*3(57), *ε*4+(16)	TC, TG, HDL, LDL
Singh, 2012 [27]	India	70	75	50–85				AD: *ε*2/*ε*2(0), *ε*2/*ε*3(4), *ε*2/*ε*4(2), *ε*3/*ε*3(23), *ε*3/*ε*4(40), *ε*4/*ε*4(1) CON: *ε*2/*ε*2(0), *ε*2/*ε*3(9), *ε*2/*ε*4(1), *ε*3/*ε*3(55), *ε*3/*ε*4(10), *ε*4/*ε*4(0)	TC, TG, HDL, LDL
Tieqiang, 2012 [28]	China	100	102	37	41.2	77.5 ± 57.3	77.0 ± 6.3	AD: *ε*2+(15), *ε*3/*ε*3(54), *ε*4+(31) CON: *ε*2+(18), *ε*3/*ε*3(70), *ε*4+(14)	TC, TG, HDL
Jie, 2013 [29]	China	157	155			71.7 ± 10.9	72.1 ± 11.5	AD: *ε*2+(20), *ε*3/*ε*3(85), *ε*4+(52) CON: *ε*2+(24), *ε*3/*ε*3(106), *ε*4+(25)	TC
Shafagoj, 2018 [14]	Jordan	38	33					AD: *APOEε4*+(11), *APOEε4*−(27)	TC, TG, HDL, LDL
Mengzhen, 2018 [30]	China	47	35	31.9	31.4	69.96 ± 8.66	68.57 ± 8.64	AD: *ε*2+(7), *ε*3/*ε*3(26), *ε*4+(14) CON: *ε*2+(7), *ε*3/*ε*3(25), *ε*4+(3)	TC, TG, HDL, LDL
Wang, 2020 [31]	China	63	33	47.6	66.7	66.3 ± 9.6	66.0 ± 8.7	AD: *APOEε4*+(28), *APOEε4*−(35) CON: *APOEε4*+(8), *APOEε4*−(25)	TC, TG, HDL, LDL

Note: APOE: apolipoprotein E; AD: Alzheimer’s disease; TC: total cholesterol (TC); TG: triglycerides; HDL: high-density lipoprotein; LDL: low-density lipoprotein; CON: healthy control population; GHP: general healthy population; *APOEε4*+: carriers of the apolipoprotein *Eε4* allele; *APOEε4*−: non-carriers of the apolipoprotein *Eε4* allele; *ε*2+: carriers of the apolipoprotein *Eε2* allele (the apolipoprotein *Eε2*/apolipoprotein *Eε4* was classified into the *ε*2+ group); *ε*4+: carriers of the apolipoprotein *Eε2* allele; *ε*2/*ε*2: apolipoprotein *Eε2*/apolipoprotein *Eε2*; *ε*2/*ε*3: apolipoprotein *Eε2*/apolipoprotein *Eε3*; *ε*2/*ε*4: apolipoprotein *Eε2*/apolipoprotein *Eε4*; *ε*3/*ε*3: apolipoprotein *Eε3*/apolipoprotein *Eε3*; *ε*3/*ε*4: apolipoprotein *Eε3*/apolipoprotein *Eε4*; *ε*4/*ε*4:apolipoprotein *Eε4*/apolipoprotein *Eε4*.

## Data Availability

The original material presented in this study is included as Supplementary Material.

## Supplementary Materials

The following supporting information can be downloaded: https://www.mdpi.com/article/10.3390/brainsci13111554/s1, Figure S1: random effect forest map of TC; Figure S2: random effect forest map of LDL; Figure S3: random effect forest map of TG; Figure S4: random effect forest map of HDL; Figure S5: sensitivity analysis for TC; Figure S6: sensitivity analysis for LDL; Figure S7: sensitivity analysis for TG; Figure S8: sensitivity analysis for HDL; Figure S9: funnel plot of TC; Figure S10: funnel plot of LDL; Figure S11: funnel plot of TG; Figure S12: funnel plot of HDL; Figure S13: fixed effect subgroup analysis of TC; Figure S14: fixed effect subgroup analysis of LDL; Figure S15: fixed effect subgroup analysis of TG; Figure S16: fixed effect subgroup analysis of HDL; Table S1: quality assessment of NOS; Table S2: Begg bias test and Egger’s test of TC; Table S3: Begg bias test and Egger’s test of LDL; Table S4: Begg bias test and Egger’s test of TG; Table S5: Begg bias test and Egger’s test of HDL; Table S6: SUCRA ranking of ApoE4 allele carrying, ApoE3/3, and ApoE4 allele carrying (TC); Table S7: SUCRA ranking of ApoE4 allele carrying, ApoE3/3, and ApoE4 allele carrying (TG); Table S8: SUCRA ranking of ApoE4 allele carrying, ApoE3/3, and ApoE4 allele carrying (LDL); Table S9: SUCRA ranking of ApoE4 allele carrying, ApoE3/3, and ApoE4 allele carrying (HDL); Table S10: SUCRA ranking of 6 genotypes (TC); Table S11: SUCRA ranking of 6 genotypes (TG).

## Abbreviations

*APOE*: apolipoprotein E; AD: Alzheimer’s disease; TC: total cholesterol; LDL: low-density lipoprotein; TG: triglycerides; HDL: high-density lipoprotein; *APOEε4* allele-C: *APOEε4* allele carriers; *APOEε4* allele-N: *APOEε4* allele non-carriers; Aβ: amyloid-β; NOS Scale: the Newcastle–Ottawa Quality Assessment Scale; SMD: standardized mean difference; CI: confidence interval; SUCRA: the cumulative ranking curve; APP: Aβ precursor protein; VLDL: very low-density lipoprotein; CNS: the central nervous system.
