# Supplementary material for: Investigation of the Relationship between Apolipoprotein E Alleles and Serum Lipids in Alzheimer’s Disease: A Meta-Analysis
Source: Brain Sci. 2023 Nov 6;13(11):1554. doi: 10.3390/brainsci13111554 (PMC10670160; doi:10.3390/brainsci13111554)
Supplement: Supplementary file 1 [file brainsci-13-01554-s001.zip › brainsci-2674525-supplementary.pdf]

Table S1: quality assessment of NOS

	Country	selection☆	comparation☆	exposure☆	total☆
Fernandes-1999	Portugal	3	0	3	6
Wehra-2000	Poland	3	2	3	8
Sheng-2000	China	3	2	3	8
Isbir-2001	Turkey	3	0	3	6
Jingbin-2002	China	3	2	3	8
Xiangyu-2002	China	3	0	3	6
Al-Shammari-2004	Kuwait	3	2	3	8
Raygani-2006	Iran	3	0	3	6
Hall-2006	India	4	0	3	7
Sabbagh-2006	America	3	2	3	8
Dongmei-2008	China	3	2	3	8
Singh-2012	India	3	2	3	8
Tieqiang-2012	China	3	2	3	8
Jie-2013	China	3	2	3	8
Shafagoj-2018	Jordan	3	2	3	8
Mengzhen-2018	China	3	2	3	8
Wang-2020	China	3	1	3	7

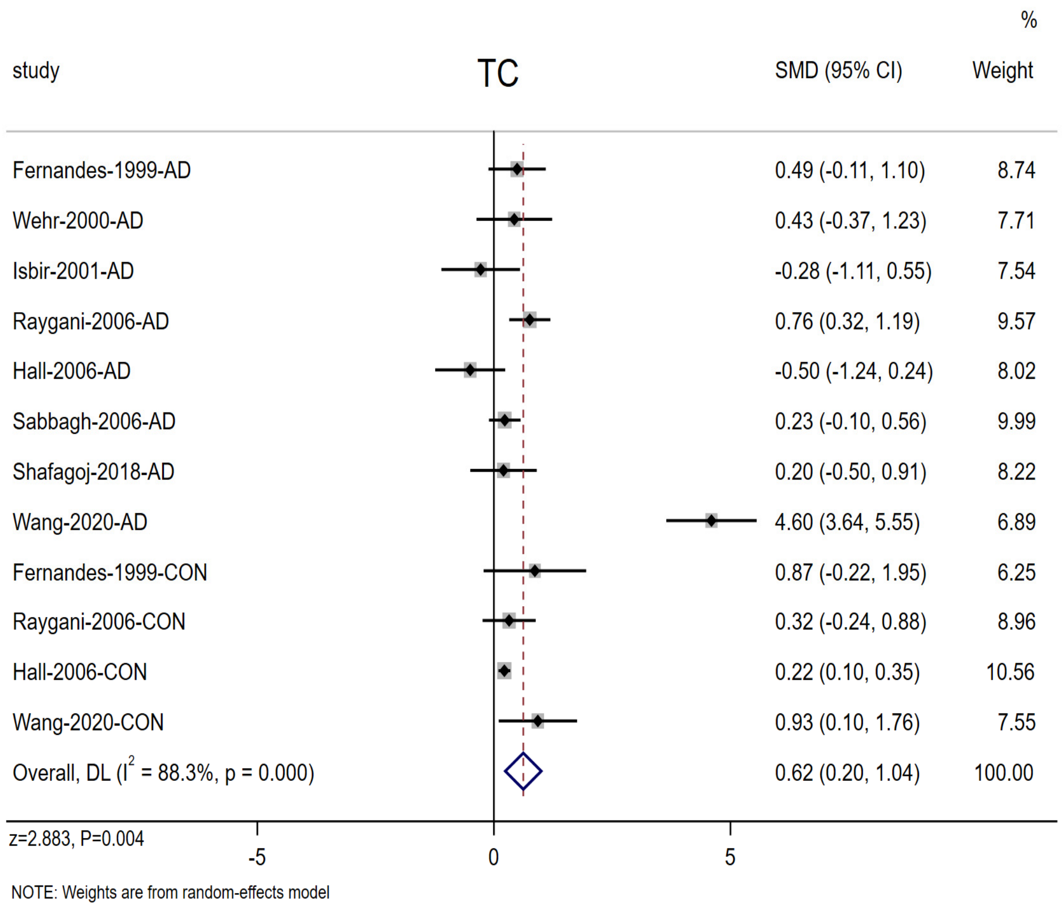

Figure S1: random effect forest map of TC

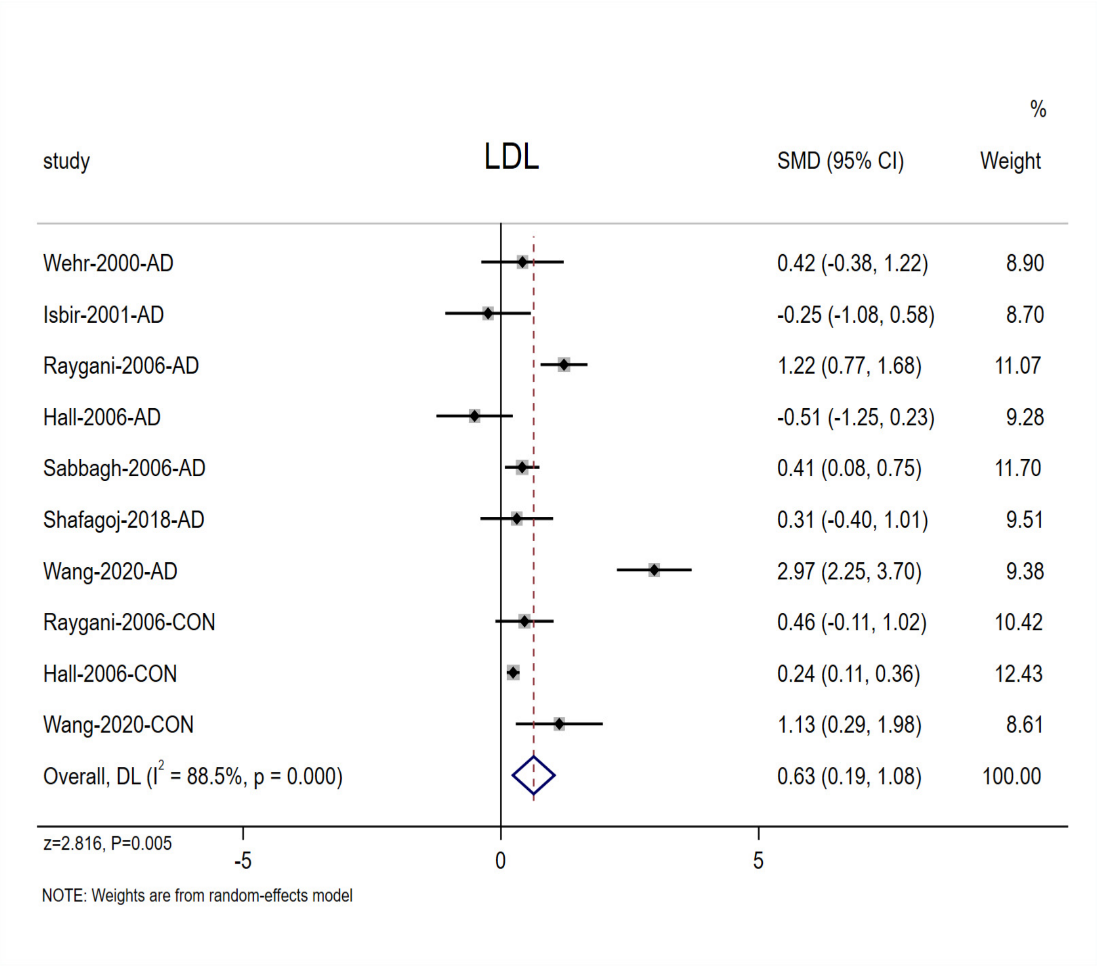

Figure S2: random effect forest map of LDL

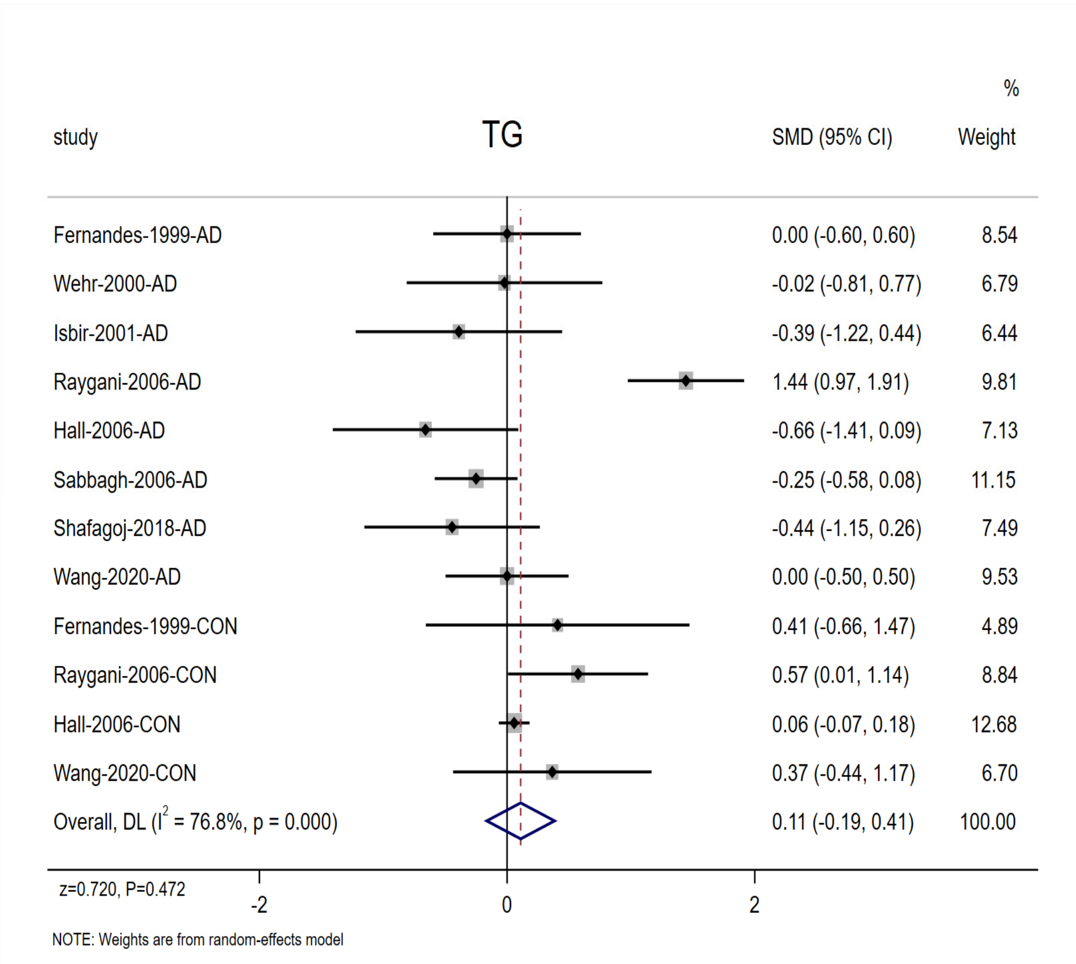

Figure S3: random effect forest map of TG

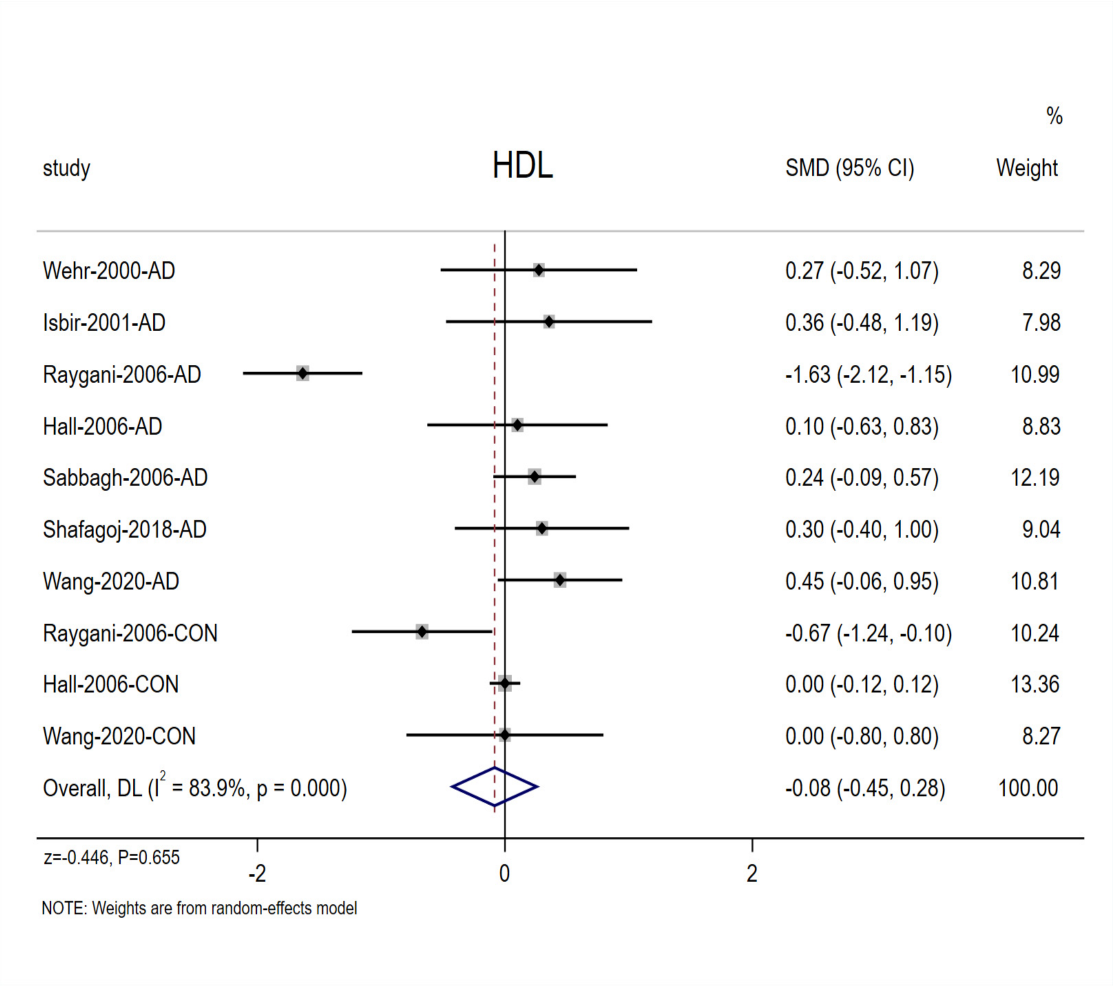

Figure S4: random effect forest map of HDL

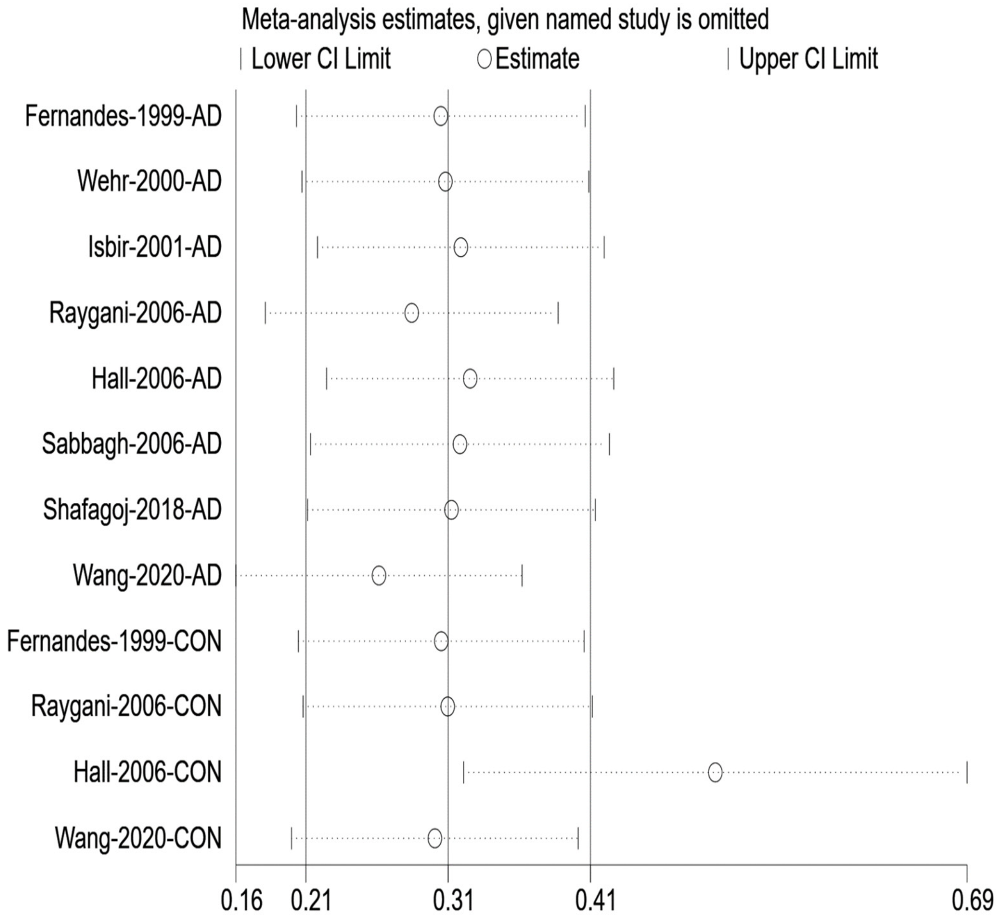

figure S5: Sensitivity analysis for TC

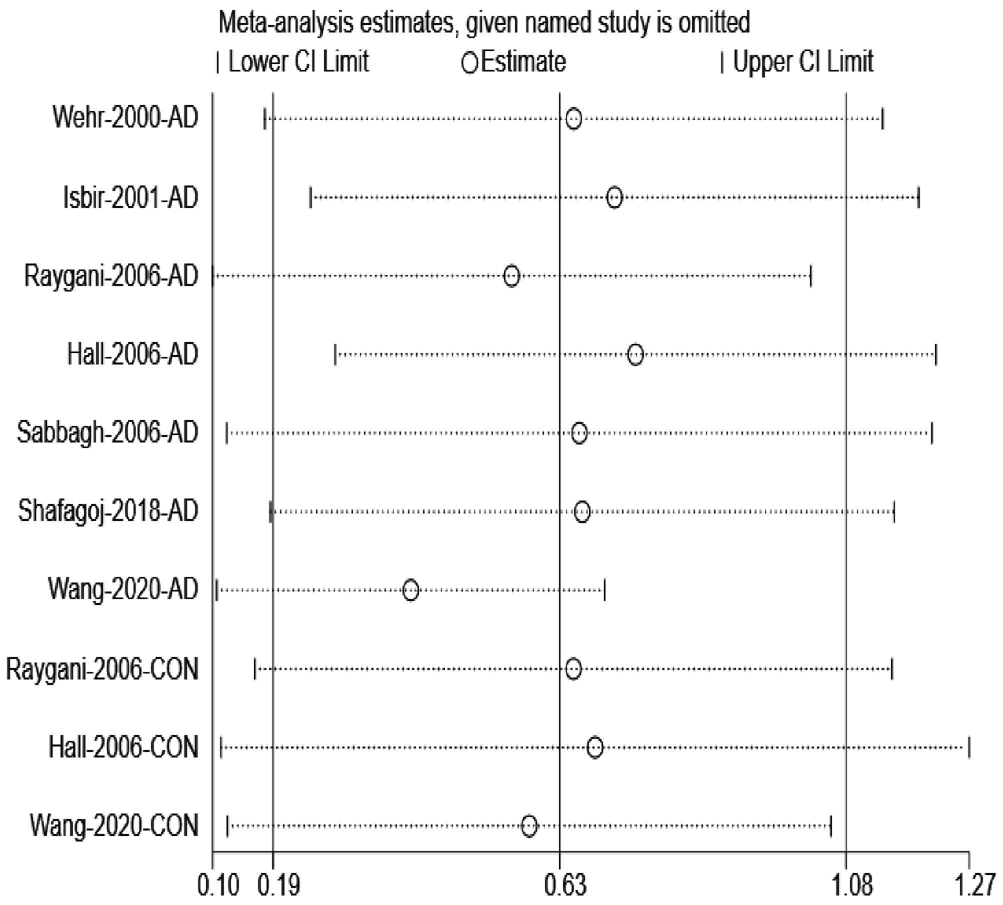

Figure S6: Sensitivity analysis for LDL

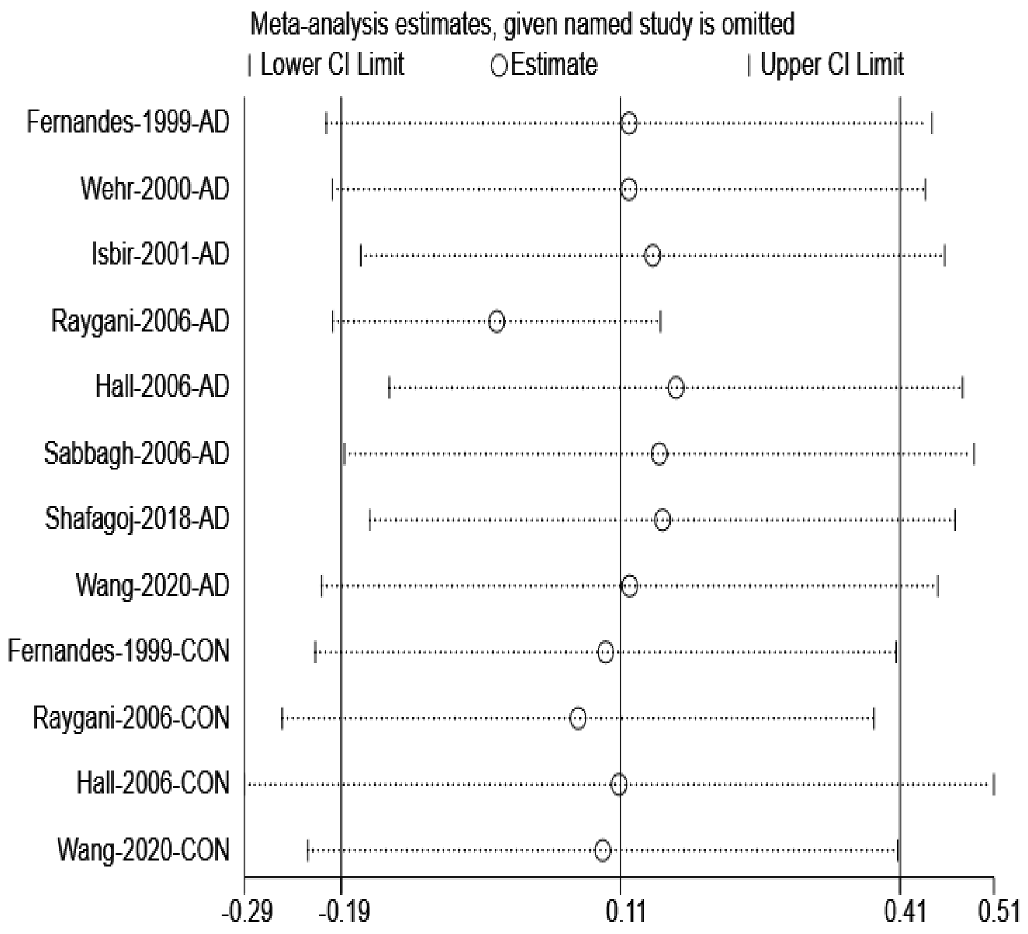

Figure S7: Sensitivity analysis for TG

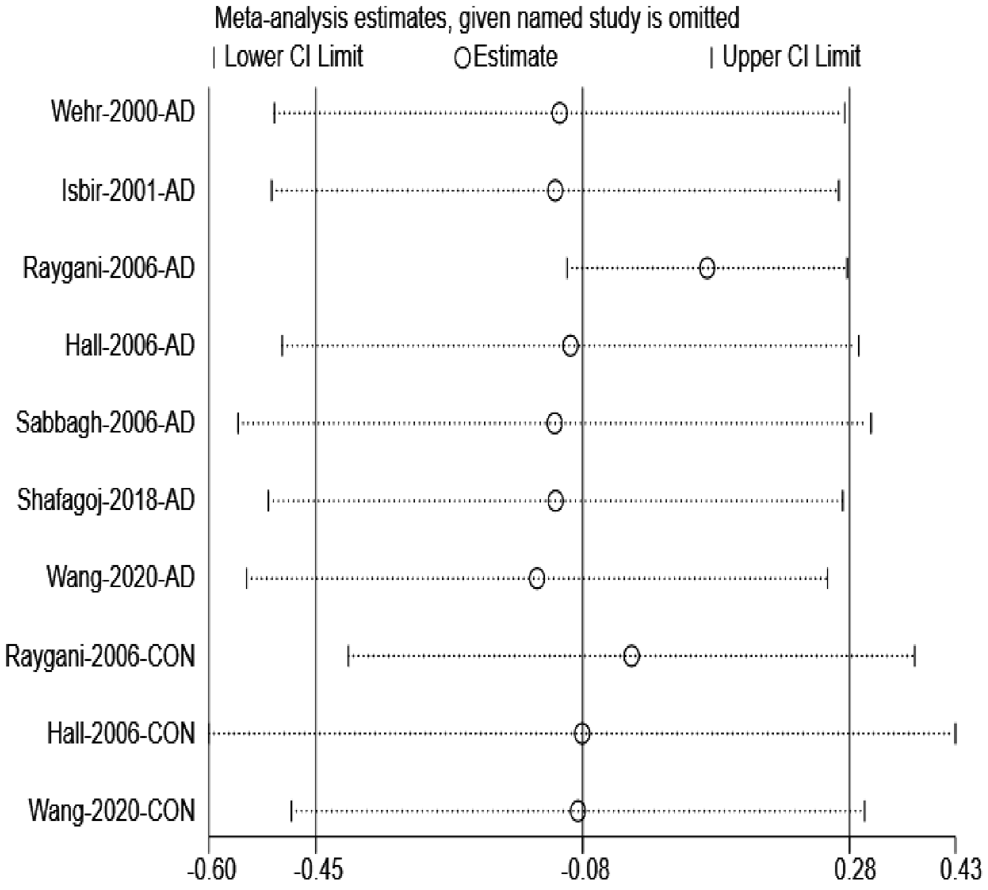

Figure S8: Sensitivity analysis for HDL

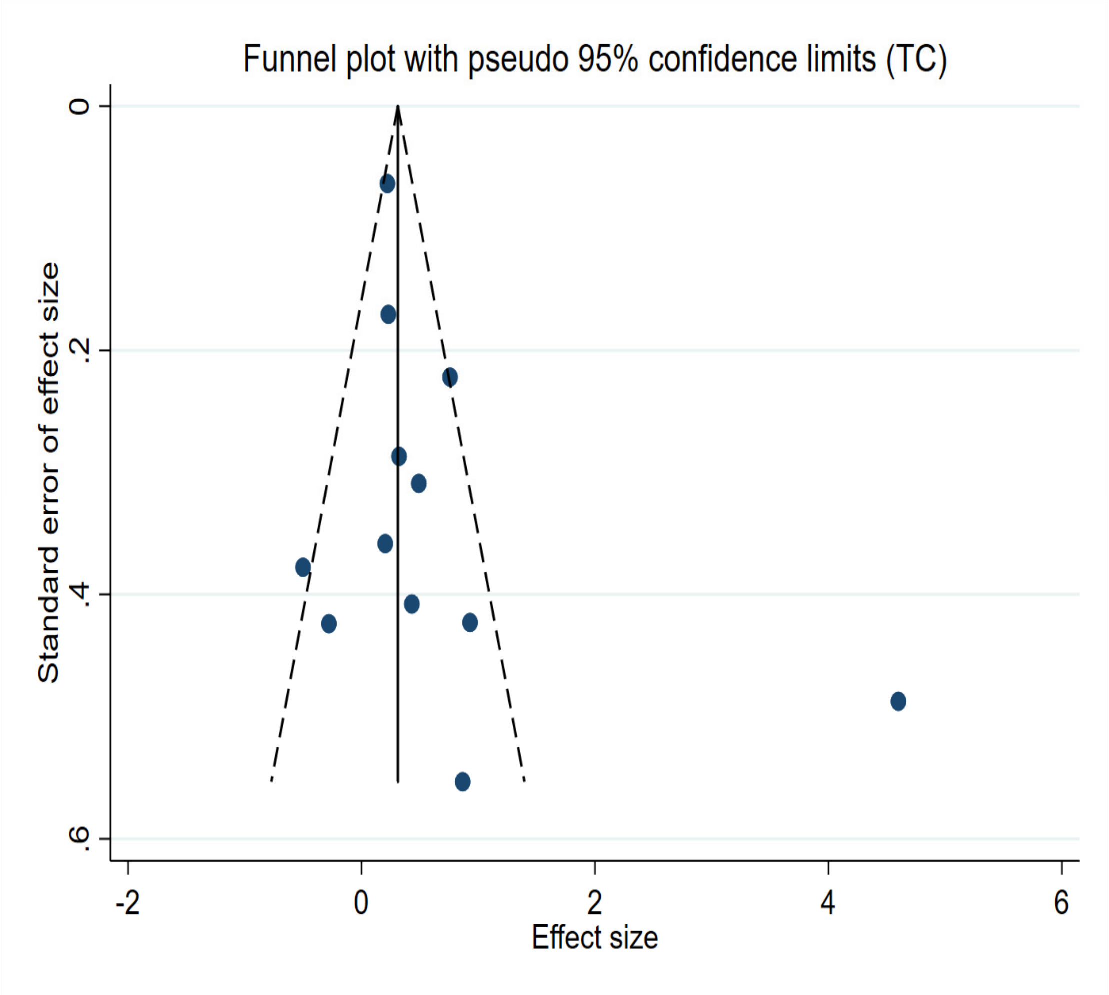

Figure S9: Funnel plot of TC

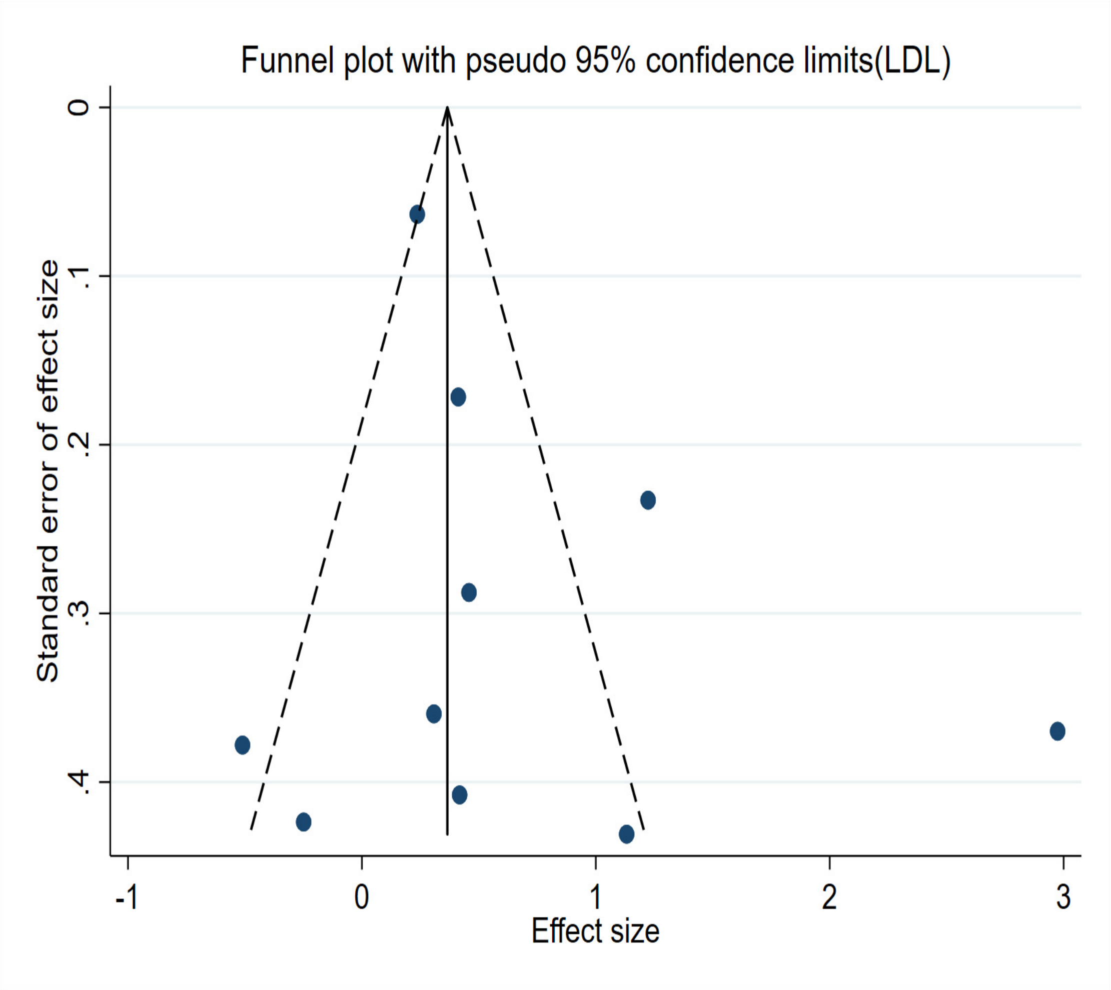

Figure S10: Funnel plot of LDL

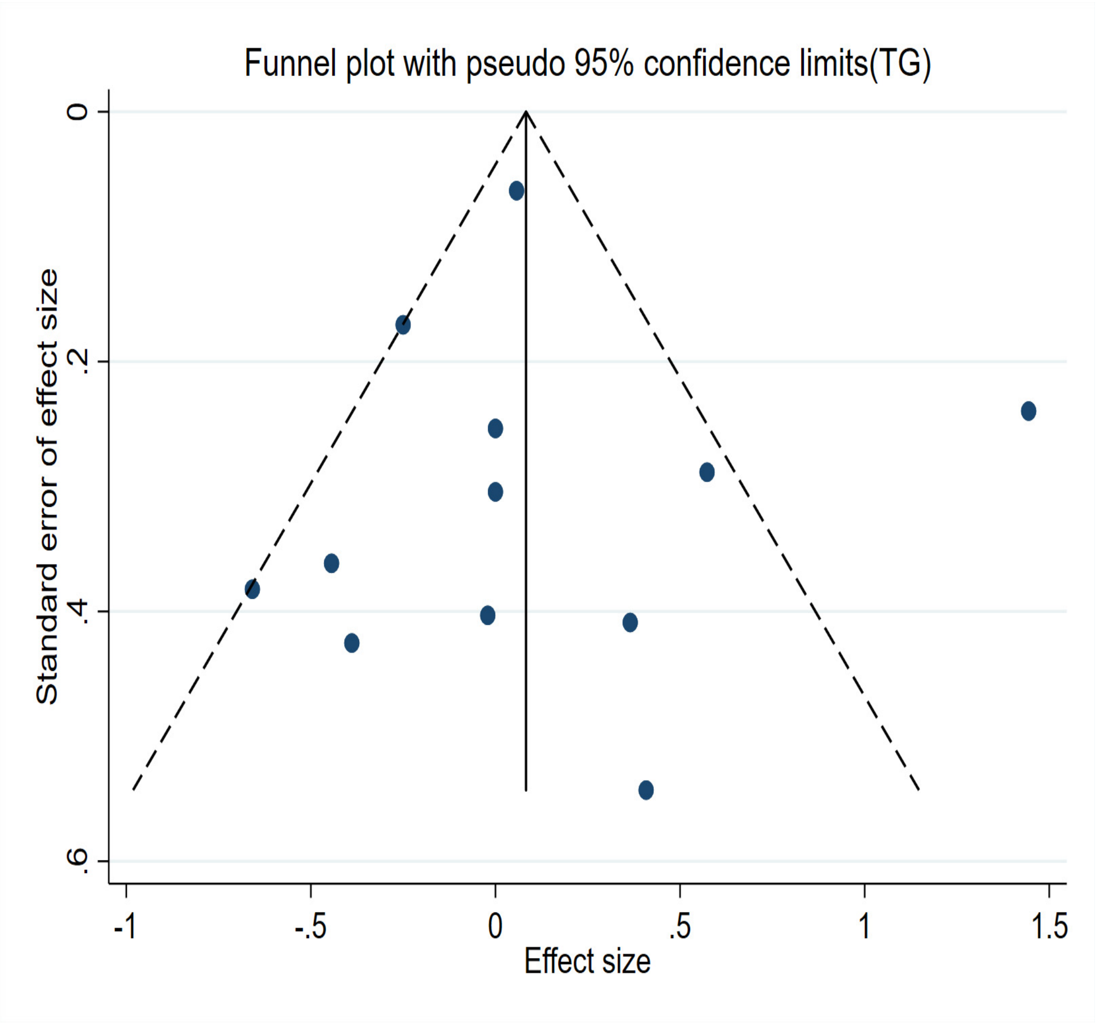

Figure S11: Funnel plot of TG

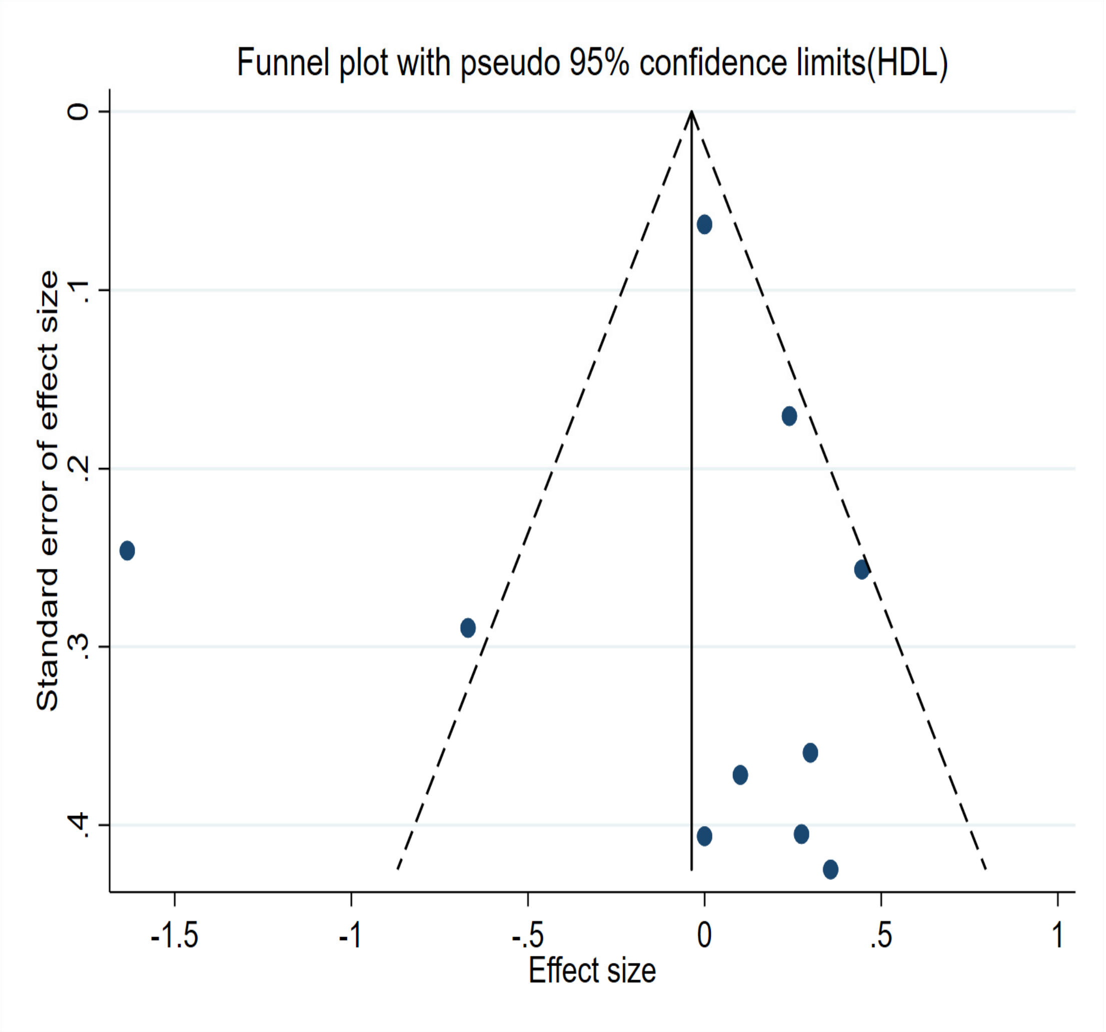

Figure S12: Funnel plot of HDL

Begg's Test

adj. Kendall's Score (P-Q) = 20
Std. Dev. of Score = 14.58
Number of Studies = 12
z = 1.37
Pr > |z| = 0.170
z = 1.30 (continuity corrected)
Pr > |z| = 0.193 (continuity corrected)

Egger's test

Std_Eff	Coef.	Std. Err.	t	P>|t|	[95% Conf	Interval]
slope	0.0986312	0.2223848	0.44	0.667	-0.3968729	0.5941353
bias	1.578166	1.228006	1.29	0.228	-1.158002	4.314334

Table S2: Begg bias test and Egger's test of TC

Begg's Test

adj. Kendall's Score (P-Q) = 3

Std. Dev. of Score = 11.18

Number of Studies = 10

z = 0.27

Pr > |z| = 0.788

z = 0.18 (continuity corrected)

Pr > |z| = 0.858 (continuity corrected)

Egger's test

Std_Eff	Coef.	Std. Err.	t	P>|t|	[95% Conf	Interval]
slope	0.1532115	0. 2323179	0.66	0.528	-0.3825146	0.6889375
bias	1.675682	1.37906	1.22	0.259	-1.504436	4.8558

Table S3: Begg bias test and Egger's test of LDL

Begg's Test

adj. Kendall's Score (P-Q) = 6

Std. Dev. of Score = 14.58

Number of Studies = 12

z = 0.41

Pr > |z| = 0.681

z = 0.34 (continuity corrected)

Pr > |z| = 0.732 (continuity corrected)

Egger's test

Std_Eff	Coef.	Std. Err.	t	P>|t|	[95% Conf	Interval]
slope	0.0573198	0.1722718	0.33	0.746	-0.3265257	0.4411654
bias	0.1877453	0.9645378	0.19	0.850	-1.961379	2.336869

Table S4: Begg bias test and Egger's test of TG

Begg's Test

adj. Kendall's Score (P-Q) = -7
Std. Dev. of Score = 11.18
Number of Studies = 10
z = -0.63
Pr > |z| = 0.531
z = 0.54 (continuity corrected)
Pr > |z| = 0.592 (continuity corrected)

Egger's test

Std_Eff	Coef.	Std. Err.	t	P>|t|	[95% Conf	Interval]
slope	0.0090646	0.2140774	0.04	0.967	-0.4845988	0.502728
bias	-0.3602882	1.286664	-0.28	0.787	-3.32734	2.606764

Table S5: Begg bias test and Egger's test of HDL

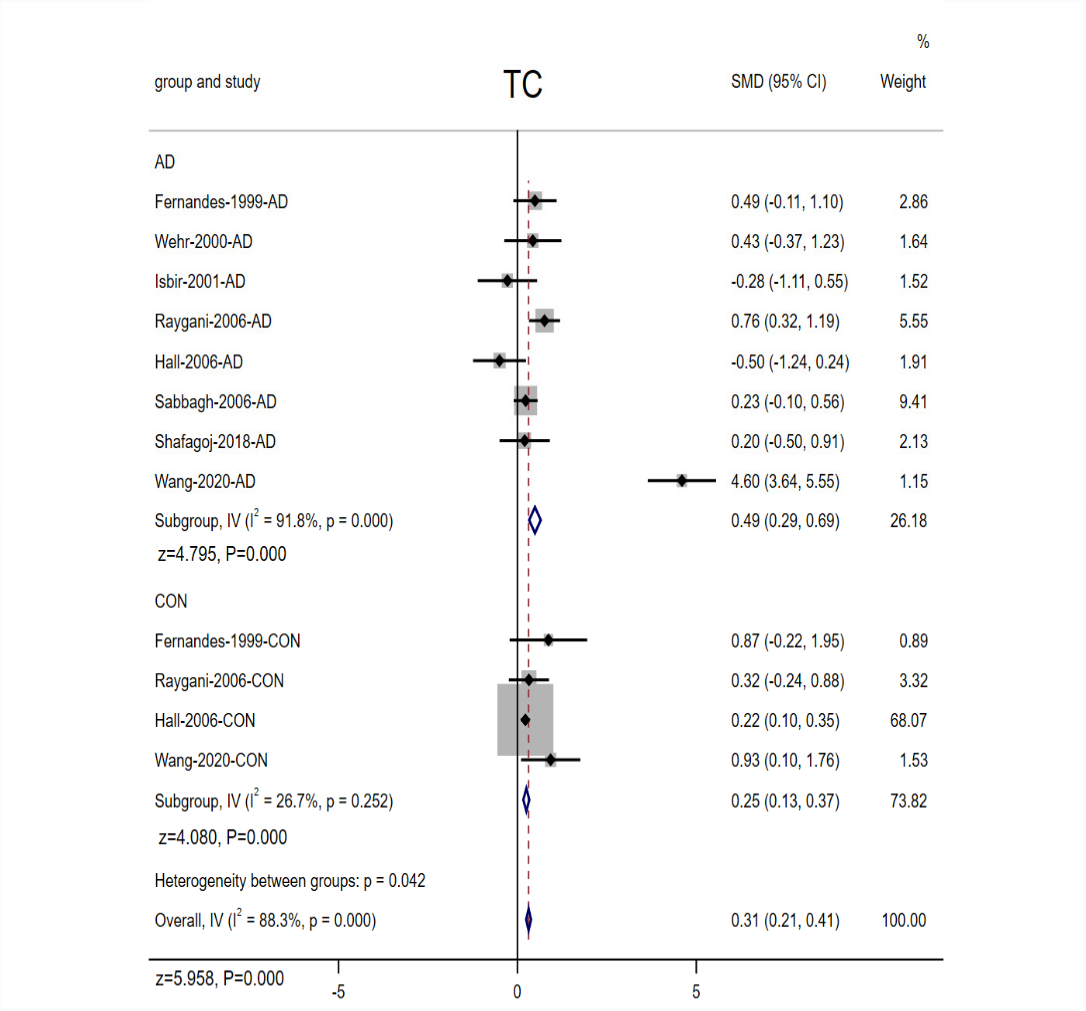

Figure S13: Fixed effect subgroup analysis of TC

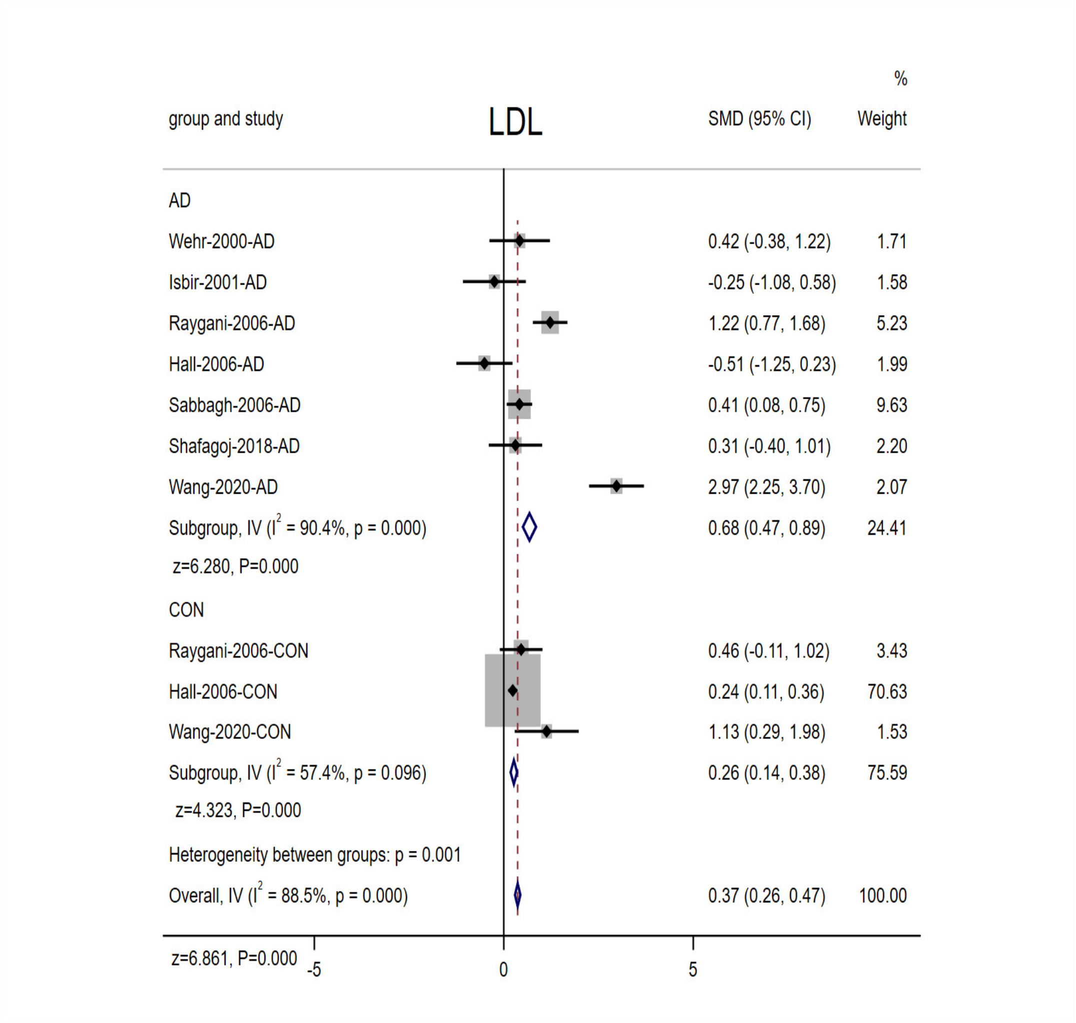

Figure S14: Fixed effect subgroup analysis of LDL

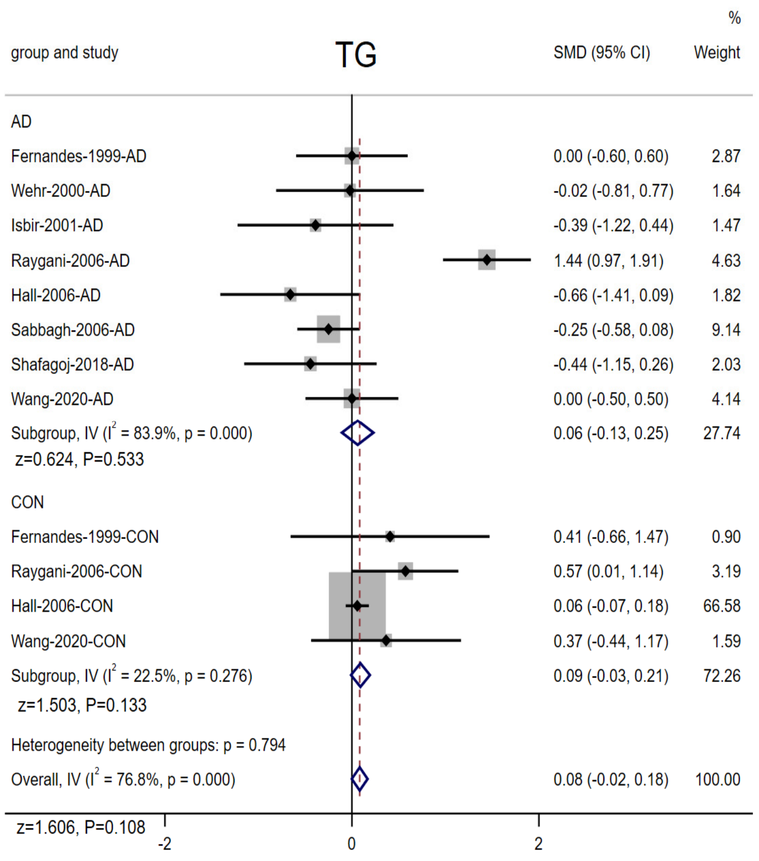

Figure S15: Fixed effect subgroup analysis of TG

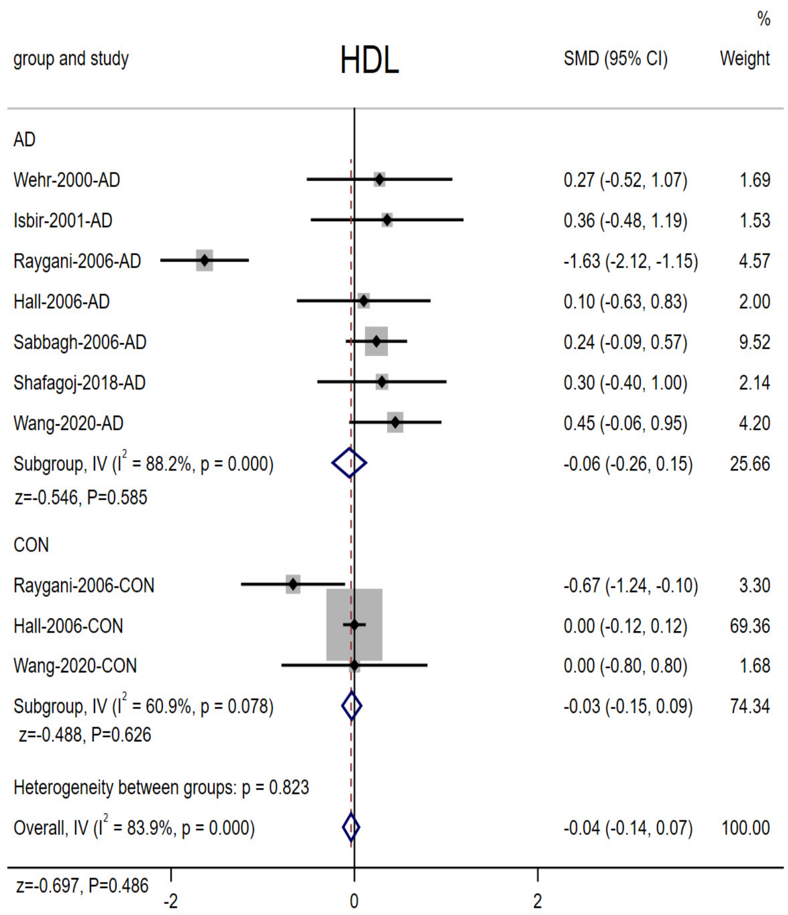

Figure S16: Fixed effect subgroup analysis of HDL

Treatment	SUCRA	PrBest	MeanRank
E2carriers	0.2	0.0	3.0
E3/E3	49.9	0.3	2.0
E4carriers	99.9	99.7	1.0

Table S6: SUCRA ranking of ApoE4 allele carrying, ApoE3/3, and ApoE4 allele carrying (TC)

Treatment	SUCRA	PrBest	MeanRank
E2carriers	17.1	3.4	2.7
E3/E3	40.7	10.1	2.2
E4carriers	92.2	86.5	1.2

Table S7: SUCRA ranking of ApoE4 allele carrying, ApoE3/3, and ApoE4 allele carrying (TG)

Treatment	SUCRA	PrBest	MeanRank
E2carriers	1.1	0.0	3.0
E3/E3	51.5	5.1	2.0
E4carriers	97.4	94.9	1.0

Table S8: SUCRA ranking of ApoE4 allele carrying, ApoE3/3, and ApoE4 allele carrying (LDL)

Treatment	SUCRA	PrBest	MeanRank
E2carriers	49.3	29.6	2
E3/E3	77.5	62.2	1.5
E4carriers	23.2	8.2	2.5

Table S9: SUCRA ranking of ApoE4 allele carrying, ApoE3/3, and ApoE4 allele carrying (HDL)

Treatment	SUCRA	PrBest	MeanRank
E2/E2	44.3	14.2	3.8
E2/E3	23.3	0.0	4.8
E2/E4	35.0	7.1	4.2
E3/E3	45.1	0.3	3.7
E3/E4	91.9	66.7	1.4
E4/E4	60.3	11.8	3.0

Table S10: SUCRA ranking of 6 genotypes (TC)

Treatment	SUCRA	PrBest	MeanRank
E2/E2	83.4	79.2	1.8
E2/E3	33.5	0.6	4.3
E2/E4	2.5	0.0	5.9
E3/E3	59.6	4.5	3.0
E3/E4	72.0	11.4	2.4
E4/E4	49.1	4.3	3.5

Table S11: SUCRA ranking of 6 genotypes (TG)
